# Supplementary material for: Isolation and Characterization of Antioxidant Peptides from Dairy Cow (Bos taurus) Placenta and Their Antioxidant Activities
Source: Antioxidants (Basel). 2024 Jul 29;13(8):913. doi: 10.3390/antiox13080913 (PMC11352039; doi:10.3390/antiox13080913)
Supplement: Supplementary file 1 [file antioxidants-13-00913-s001.zip › antioxidants-3099231-supplementary.pdf]

Table S1 Primers used for real-time qPCR assay

**Primer name**	**Sequence (5'to3')**	**Transcript ID**
Keap1	F: CAGATTGACAGCGTGGTTCG<br>R: TGCGGGCAGTCGTATTTGA	NM_001110305.1
Nrf2	F: TGTCTTAATACCGAAAACAAGCAGC<br>R: GACCACAGTTGCCCACTTCTTTT	NM_010902.5
HO-1	F: GCTAAGACCGCCTTCCTGCT<br>R: ACGAAGTGACGCCATCTGTGA	NM_001317450.2
GAPDH	F: CCTCGTCCCGTAGACAAAATG<br>R: TGAGGTCAATGAAGGGGTCGT	NM_001289726.2

Table S2 Antioxidant activity, water solubility, stability, sensitization and toxicity of 129 peptides.

Polypeptide	Antioxidant activity	Stability	Water solubility	Sensitizatio
AEEGIAAGGVMDVNTALQEVLK	–	41.97	0.318	+
AELTAPFPPVG	+	88.92	0.455	–
AKPQHVTETFHPDPNWKNFQKPGT	–	2.49	-1.554	+
ALMLQGVDLLADAVAVTMGPK	–	9.8	1.014	+
ALQEASEAYLVGLFEDTNLCAIHAK	–	35.27	0.276	+
AMGIMNSFVNDIFER	–	3.67	0.313	–
ANNGKQWAEVF	+	-4.43	-0.782	–
AQLGVQAFADALLIIPK	+	2.72	1.071	–
ARGSDGSVGPVGPAGPIG	+	16.69	-0.017	+
DDDIAALVVDNGSGMCK	+	-8.27	0.1	–
DDNDEDGDDLLH	+	25.22	-2	–
DEQNEEKRR	+	166.38	-3.767	–
DEQNEEKRRQ	+	170	-3.74	–
DFGHIQYVAAYR	+	19.12	-0.217	+
DGLDGEDGDKG	+	4.31	-1.745	+

DGRHDPRDDDLNL	+	18.25	-2.123	–
DGRHDPRDDDLNLR	+	31.41	-2.293	–
DLFENTNHTQVQ	+	-29.28	-1.233	–
DLYANTVLSGGTTMYPGIADR	–	24.07	-0.071	+
DNLLDDLQR	+	45.11	-1.178	+
DVDIIDHHDNTYTVK	+	-13.93	-0.873	+
EEDIRPYIS	+	87.91	-1.078	–
EEDIRPYISR	+	99.38	-1.42	+
EEEIAALVIDNGSGMCK	+	50.25	0.118	+
EFQRIARRDKKAFFSDQCKEIE	–	57.02	-1.227	–
ESEIIDFFLGASLK	+	33.04	0.543	–
ESIQELHASDMPEYSVT	+	63.15	-0.618	–
ESIQELHASDMPEYSVTN	+	51.84	-0.778	–
ETLHDAMH	+	80.35	-0.825	+
EVAAFAQFGSDLDAATQQLLSR	–	20.2	0.091	+
EVEPSDTIENVK	+	62.96	-0.967	–
FDENDVITCFANFESDEVELSYAK	–	26.35	-0.325	–

FETFNVPAM	+	65.4	0.467	+
FGFDGDFY	+	55.5	-0.087	+
FGFDGDFYR	+	31.66	-0.578	–
FGTIGIGTPAQD	+	20.04	0.2	+
FGTIGIGTPAQDF	+	13.47	0.4	–
FNQTDLTIK	–	-8.92	-0.522	–
FTPEELEKYQ	+	58.98	-1.49	+
GFGFVTYATVEEVDAAMNAR	–	25.88	0.265	–
GKGLVGAVT	+	-38.28	0.911	–
GPAGPIGPVG	+	39.03	0.41	+
GPGGLPGFYAQ	+	88.98	-0.109	+
GPGGSVDSGPPPDR	+	37.86	-1.207	–
GPLPMEAIEKMAS	+	86.62	0.031	+
GPPGPEGPPGAPGPEGP	+	58.85	-1.2	+
GPPGTPGSPG	+	63.71	-0.95	–
GPPLSQSQKWLPS	+	126.6	-0.908	+
HDPRDDDLNLR	+	19.75	-2.155	+

HLNDDVVK	+	5.15	-0.675	–
HYDDSMPDPLPE	+	142.43	-1.533	+
IINIFHQYSVR	–	42.18	0.336	+
IITITGTQDQIQNAQYLLQNSVK	–	16.92	-0.213	–
ILSISADIETIGEILK	+	57.63	0.925	+
INALTAASEAACLIVSVDETIK	–	32.48	0.914	+
ISEAEGPPGGSGSQGSSSPSQ	–	100.52	-0.957	–
ISQEEAGAGAGEQTCAPGSRPPDMR	–	60.73	-0.904	–
ISQEEMEFFTTSSGNT	–	55.25	-0.65	–
ITGLDPAGPNFEYAEAPSR	+	50.11	-0.547	–
KEEEDKDDEEKPK	+	86.95	-3.477	–
KGNYLILK	+	0.99	-0.113	+
KKYDEELEERLVE	+	77.6	-1.754	+
KPEYDEAGPSIVH	+	43.3	-0.985	–
KPLVIIAEDVDGEALSTLVLNR	–	4.61	0.5	–
KSPEELKGIFEKYAAK	+	66.96	-0.969	–
KVIDELDVKPE	+	7.7	-0.609	+

LCYVALDFEQEMATAASSSSLEK	–	53.52	0.148	–
LEVEPSDTIENVK	+	58.88	-0.6	–
LEVEPSDTIENVKAK	+	52.37	-0.66	–
LFIGGLSFETTDESLR	+	41.16	0.169	–
LFIGGLSFETTEESLR	+	73.58	0.169	+
LGFSEVELVQMVVDGVK	+	10.21	0.812	+
LHEEQGEIILK	+	76.68	-0.445	+
LNEQGLR	+	63.6	-1.114	–
LNEQGLRDIANTPHELYR	+	16.73	-1.083	+
LSEQEMSSAREEACVRIQQALTMFP	–	101	-0.296	–
MPMWDEEEDEEAK	–	127.28	-1.946	–
MRPDSTEIDQDTIN	–	56.56	-1.314	+
MTQSLLENEALNLKNQSLARQTDPS	–	49.58	-0.832	–
NAPAIIFIDELDAIAPK	+	43.4	0.629	+
NDFQLIGIQDGYLSLLQDSGEVR	–	34.92	-0.235	–
NGKTSDYLLLG	+	-12.29	-0.282	+
NLQTDLSDGLR	+	56.24	-0.818	+

NWDDMEKIW	–	-12.07	-1.478	+
PQFYELLIK	+	40.32	0.122	–
QEASFTITVPPSA	+	68.49	0.146	+
QESLENPMPCWLYLGD	+	72.2	-0.519	–
QIKQVEDDIQQLL	–	44.75	-0.585	–
QPRWSSSSLDGNGCTDTTMCPAYAT	–	82.81	-0.656	+
RDNLLDDLQR	+	41.6	-1.51	+
RDNLLDDLQRLK	+	29.26	-1.267	–
REEYLLLK	+	38.89	-0.662	+
RHSQTTDDPQ	+	66.78	-2.55	–
RNDEELNK	+	94.53	-2.763	–
SAAEMYGSSFDLDYDFQR	+	44.41	-0.694	+
SGETEDTFIADLVVGLCTGQIK	–	21.79	0.3	–
SLQDIIAILGMDELSEEDKLTVSR	–	69.8	0.008	–
SPEELKGIFEKYAAK	+	70.75	-0.773	–
SPGDELYKIIQ	+	41.32	-0.518	+
SQLEEKENKKFPVF	+	90.76	-1.286	–

SRFDQHQNKPE	+	68.29	-2.609	–
SRFDQHQNKPEIL	+	88.95	-1.569	+
SRFDQHQNKPEILN	+	83.31	-1.707	+
SRFDQHQNKPEILNLVK	+	68.68	-1.165	+
STAISLFYELSENDLNFIK	+	20.42	0.105	–
TAELTAPFPPVG	+	82.34	0.358	–
TALLDAAGVASLLTTAEVVVTEIPK	–	20.46	1.02	+
TDDHTLIR	–	-0.68	-0.975	–
TDEDTIID	–	14.04	-0.8	–
TDIDKDSNTDIL	–	27.12	-0.9	+
TIGGGDDSFNTFFSETGAGK	–	30.29	-0.445	–
TITLEVEPSDTIENVK	+	49.72	-0.294	–
TLVLSNLSYSATEETLQEVFEK	–	56.69	-0.114	–
TNWDDMEKIW	–	-24.89	-1.4	+
TSQEDRQLIN	+	78.5	-1.52	–
TVTAMDVVYALK	–	8.47	8.47	+
VEGPPGPEGPA	+	59.91	-0.782	+

VEGPPGPEGPAG	+	55.75	-0.75	+
VETGVLKPGMVVTFAPVNVTTEVK	–	20.31	0.608	+
VGEAGPEGPPGEP	+	34.1	-0.962	+
VLEYLTAEILELAGNAAR	+	26.13	0.567	–
VPPPFPPIQ	+	137.29	0	–
WELTDDKNQRF	+	-5.48	-1.9	+
WELTDDKNQRFF	+	-4.19	-1.508	–
WGDAGAEYVVESTGVFTTMEK	–	5.67	-0.157	–
YDCGEEILITVLSAMTEEAAVAIK	–	62.81	0.658	–
YYAPFDGIL	+	117.39	0.533	+
YYAPFDGILG	+	106.65	0.44	–

Table S3 Mass spectrometry identification results of R2 component

Peptide	-10lgP	Mass	Length	Area
Q(+42.01)PGLPGPAG	22.64	834.4235	9	1.03E+08
ENIRPFIS	20.14	974.5185	8	2.63E+07
RFKDLGE	20.13	863.4501	7	1.80E+07
RFKDIGE	20.13	863.4501	7	1.80E+07
GPGEVLPR	29.38	823.4551	8	1.72E+07
VPPPFPPIQ	25.57	990.5538	9	1.30E+07
AGDWSVH	24.9	770.3347	7	1.11E+07
GPIGNVGAP	25.02	780.413	9	4.77E+06
GPVC(+57.02)PFR	21.8	831.4061	7	4.22E+06
SC(+57.02)LEEFR	21.68	939.412	7	2.78E+06
ENIFFAH	25.21	876.413	7	2.18E+06
SC(+57.02)LEDFR	22.98	925.3964	7	1.94E+06
AATPPRGPAE	24.75	965.493	10	1.61E+06
TPNYEVK	22.26	849.4232	7	1.52E+06
DGSINFR	26.5	807.3875	7	1.11E+06
GDLTDFLK	20.85	907.4651	8	1.05E+06
PAALPSGPAGE	24.46	965.4818	11	9.90E+05
TWNSGALK	26.51	875.4501	8	6.76E+05
DEGIRYR	21.11	907.4512	7	6.01E+05
PDTEKQIK	30.26	957.5131	8	5.98E+05
GPVPPVR	22.44	720.4282	7	0

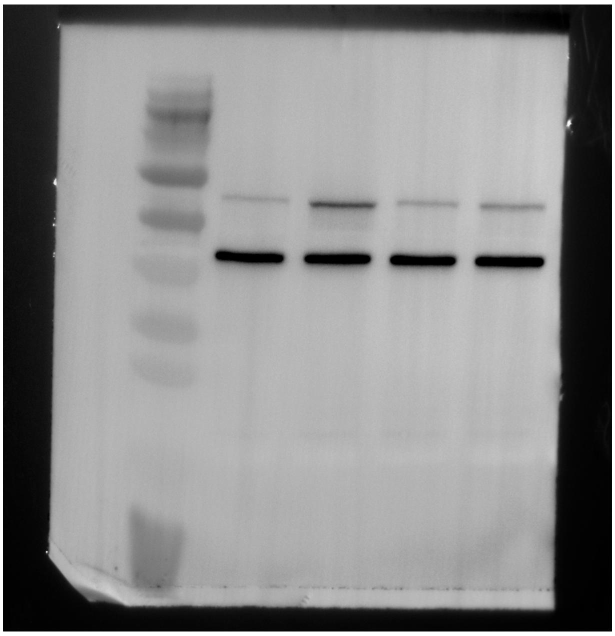

Figure S1. Detection of Keap1 protein expression

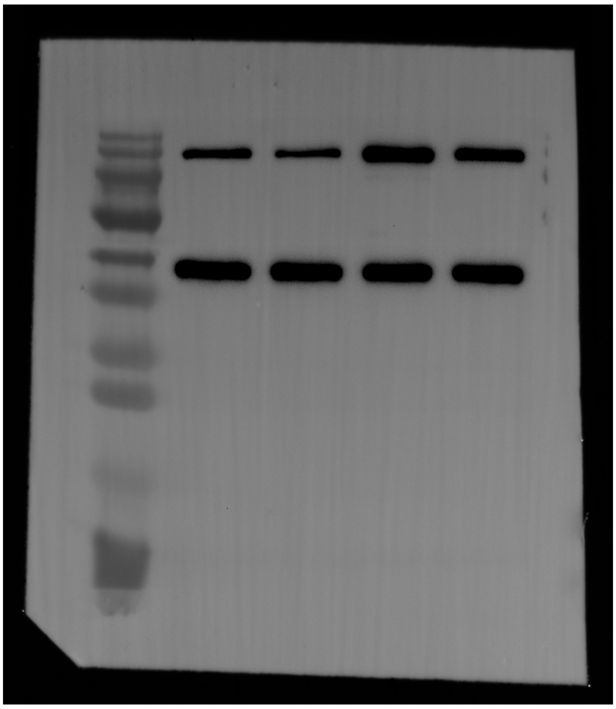

Figure S2. Detection of Nrf2 protein expression

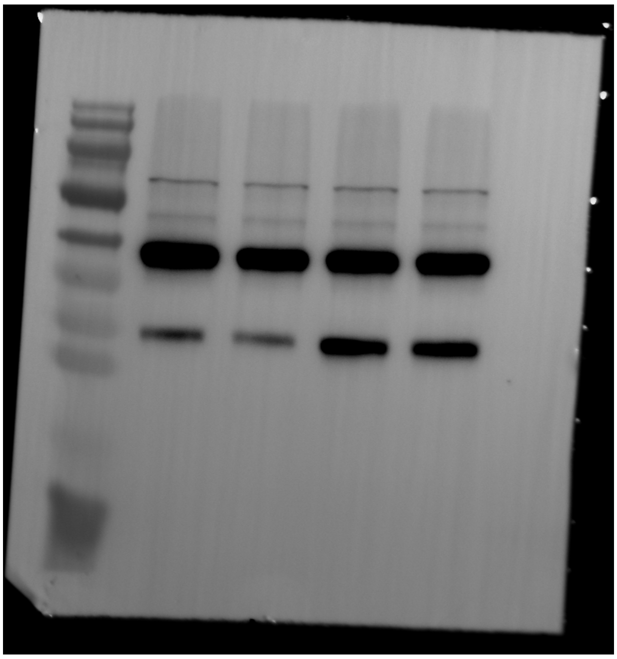

Figure S3. Detection of HO-1 protein expression
